# The potential value of fibrinogen-to-albumin ratio in assessing disease activity in rheumatoid arthritis

**DOI:** 10.3389/fimmu.2025.1670731

**Published:** 2025-11-18

**Authors:** Xiaoli Li, Jingfang Shen, Lina Leng, Yaorong Han, Jinfeng Zhang, Lianju Li, Yuhua Qiao

**Affiliations:** 1 Department of Rheumatology, Xingtai People’s Hospital, Xingtai, Hebei, China; 2 Department of Surgical Urology, Xingtai People’s Hospital, Xingtai, Hebei, China

**Keywords:** fibrinogen to albumin ratio, disease activity score 28, rheumatoid arthritis, non-linear association, a cross-sectional study

## Abstract

**Background:**

The fibrinogen-to-albumin ratio (FAR) has emerged as a promising inflammatory marker, but its relationship with rheumatoid arthritis (RA) disease activity remains unclear. This study sought to elucidate the association between FAR and RA disease activity and to assess its potential for identifying high disease activity states.

**Methods:**

We conducted a cross-sectional study of 1,191 consecutive RA inpatients at Xingtai People’s Hospital from March 2022 to December 2024. FAR was calculated as fibrinogen (g/L) divided by albumin (g/L). Disease activity was assessed using the 28-joint Disease Activity Score (DAS28) based on C-reactive protein (DAS28-CRP) and erythrocyte sedimentation rate (DAS28-ESR). Multiple linear regression and generalized additive models were employed to examine the association of FAR with disease activity. Receiver operating characteristic (ROC) analysis evaluated FAR’s discriminatory performance for high disease activity.

**Results:**

After exclusions, 981 patients (mean age, 57.7 years; 77.9% female) were included; 95.6% had moderate-to-high disease activity. A significant nonlinear association between FAR and disease activity was detected, with a saturation threshold at FAR = 0.14. Below this threshold, FAR was strongly and positively associated with DAS28-ESR [β = 15.21; 95% confidence interval (CI), 13.01–17.42] and DAS28-CRP (β = 13.07; 95% CI, 11.07–15.07). Above the threshold, associations were substantially attenuated and not statistically significant (DAS28-ESR: β = 2.19; 95% CI, −1.53–5.92; DAS28-CRP: β = 3.37; 95% CI, −0.01–6.74). Furthermore, FAR demonstrated good discriminatory ability between high and moderate disease activity, with area under the curve (AUC) values of 0.80 for DAS28-ESR and 0.81 for DAS28-CRP.

**Conclusion:**

This study identified a nonlinear relationship between FAR and RA disease activity in inpatients with predominantly moderate-to-high disease activity, characterized by a saturation threshold effect. FAR showed good discriminatory ability for distinguishing high from moderate disease activity. These findings suggest that FAR may serve as a promising and readily accessible inflammatory marker to complement existing assessments of disease activity. However, multicenter validation is warranted.

## 1 Introduction

Rheumatoid arthritis (RA) is a chronic, systemic autoimmune disease characterized by persistent inflammation, polyarticular involvement, and synovitis (1). Beyond joint damage, RA may affect extra-articular tissues such as the lungs, hematologic system, and skin, leading to significant impairment in quality of life and imposing a considerable economic burden on patients and society (1–3). Globally, approximately 1% of the population is affected by RA, with the number of cases expected to reach 31.7 million by 2050 (4, 5).

Although the exact cause of RA remains unclear, persistent chronic inflammation drives synovial proliferation, which gradually damages nearby bone and cartilage, eventually causing joint deformity and disability (1). Thus, early diagnosis and accurate assessment of disease activity are essential for improving long-term clinical outcomes. Pro-inflammatory cytokines, such as interleukin-6 (IL-6) and tumor necrosis factor α (TNF-α), exert their effects by upregulating molecules including receptor activator of nuclear factor-κB ligand, matrix metalloproteinases, and prostaglandins, collectively contributing to joint structural damage (2, 3). As a result, targeted therapies against these cytokines have been developed, significantly enhancing longitudinal outcomes for individuals with RA (3, 6). However, routine clinical measurement of cytokines such as IL-6 and TNF-α remains limited by the need for specialized and costly assays. Serum C-reactive protein (CRP) and erythrocyte sedimentation rate (ESR) are widely recognized laboratory biomarkers for evaluating inflammation in RA, but questions persist regarding their sensitivity and specificity in accurately reflecting disease activity (7). Advanced imaging techniques such as ultrasonography and magnetic resonance imaging offer valuable insights into disease status but are constrained by high costs and technical demands (8).

Recently, the fibrinogen-to-albumin ratio (FAR) has garnered increasing attention as a composite biomarker that integrates two clinically significant parameters. Fibrinogen, an acute-phase reactant, rises during inflammatory states and plays key roles in both coagulation and immune regulation (9–11). In contrast, albumin is recognized as an anti-inflammatory protein and an indicator of nutritional status, with decreased levels often reflecting chronic inflammation and oxidative stress (12). Accumulating evidence supports the clinical value of FAR across a range of inflammatory and malignant diseases, including systemic lupus erythematosus (SLE), ankylosing spondylitis (AS), inflammatory bowel disease, cardiovascular disease, and various cancers (13–18). Notably, FAR has demonstrated a positive correlation with disease activity such as the Bath Ankylosing Spondylitis Disease Activity Index in AS, outperforming individual inflammatory markers like CRP and ESR in diagnostic accuracy (16, 17). Similarly, FAR has been identified as a prognostic biomarker for disease severity and poor outcomes in SLE (15).

Despite these encouraging findings, the relationship between FAR and RA disease activity has not been thoroughly investigated. Accordingly, the present study seeks to clarify this association and to assess the discriminatory power of FAR in identifying high disease activity states.

## 2 Materials and methods

### 2.1 Study population

**Figure 1:**
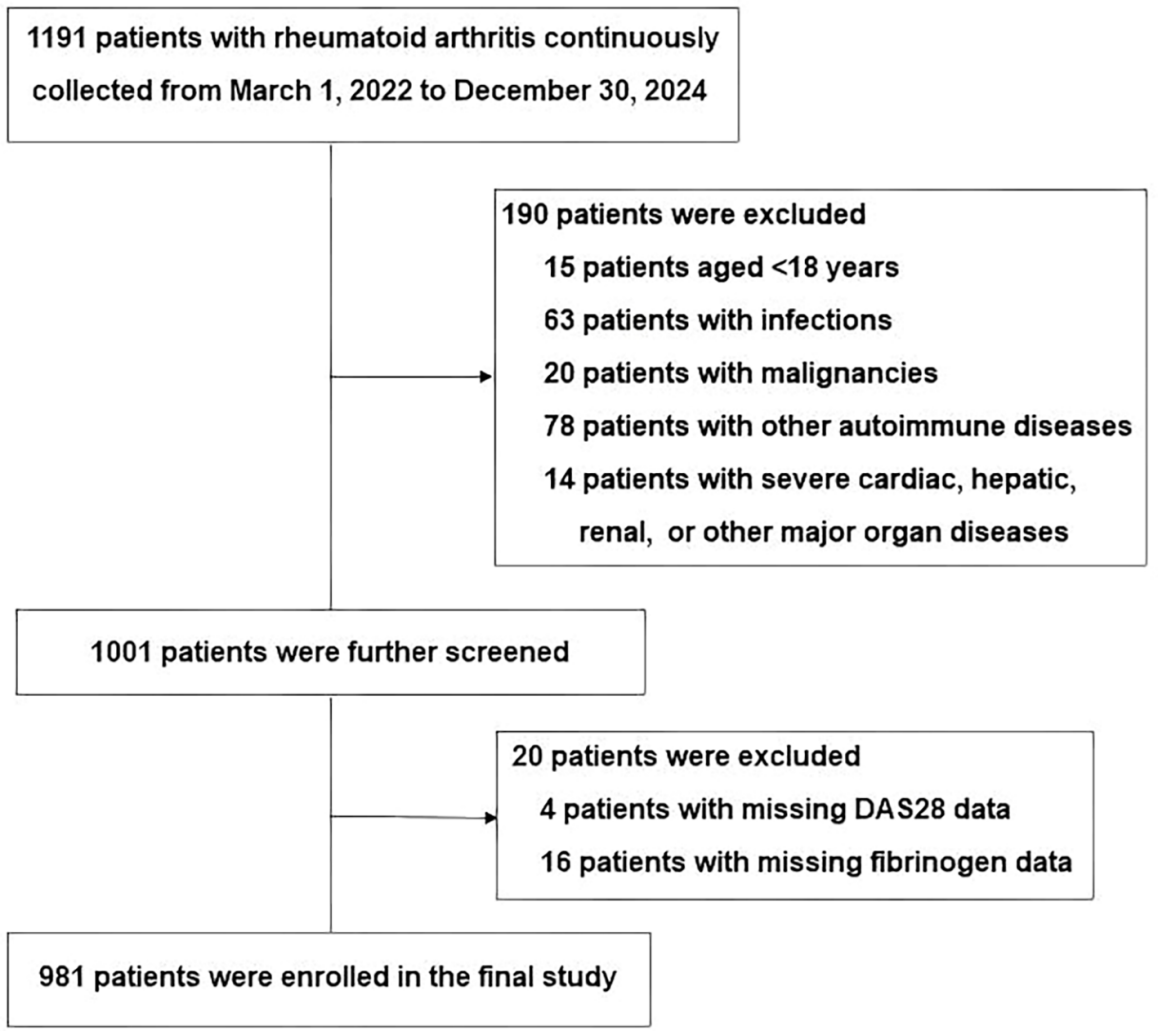
A flowchart of the screening process for patients with RA.

This cross-sectional study consecutively enrolled 1,191 inpatients with RA from Xingtai People’s Hospital between March 2022 and December 2024. All participants met the 2010 ACR/EULAR classification criteria for RA (19). Exclusion criteria included being under 18 years old, having active or chronic infections, other autoimmune conditions, severe liver or kidney dysfunction, malignancies, or missing DAS28 or fibrinogen data. After applying these criteria, a total of 981 eligible patients were retained for analysis. The study enrollment procedure is depicted in **Figure 1**. The study was conducted in accordance with the Declaration of Helsinki and approved by the Ethics Committee of Xingtai People’s Hospital (approval number: 2025[031]). Given the retrospective design of the study and the anonymization of patient data, the requirement for informed consent was waived by the Ethics Committee, consistent with previous reports (20, 21).

### 2.2 Clinical data collection

Demographic and clinical data were collected, including age, sex, body mass index (BMI), smoking status, history of alcohol consumption, and past medical history [coronary heart disease (CHD), diabetes mellitus (DM), and hypertension]. Information on RA disease duration and recent medication use [including conventional synthetic disease-modifying antirheumatic drugs (csDMARDs), targeted synthetic DMARDs (tsDMARDs), biological DMARDs (bDMARDs), glucocorticoids, and nonsteroidal anti-inflammatory drugs (NSAIDs)] was also recorded. Additionally, swollen joint count in 28 joints (SJC28), tender joint count in 28 joints (TJC28), and patient global health assessment (GH) measured by a 100-mm visual analogue scale (VAS) were documented. All data were extracted from the hospital’s electronic medical records, which were originally collected through face-to-face interviews and physical examinations performed by rheumatologists on the day of admission.

Laboratory data were obtained from the electronic medical record system and included complete blood count parameters [including white blood cell (WBC) count, red blood cell (RBC) count, platelet count, lymphocyte count, neutrophil count, and hemoglobin], liver function tests [alanine aminotransferase (ALT) and aspartate aminotransferase (AST)], ESR, CRP, rheumatoid factor (RF), TNF-α, IL-6, serum albumin, and autoantibodies such as anti-cyclic citrullinated peptide antibody (ACPA), anti-perinuclear factor (APF), and anti-keratin antibody (AKA). All laboratory tests were performed in the hospital laboratory following standardized protocols, using fasting peripheral blood samples collected on the day of admission or the following day.

Finally, the FAR was calculated by dividing fibrinogen (g/L) by albumin (g/L). The neutrophil-to-lymphocyte ratio (NLR) was calculated as neutrophil count divided by lymphocyte count, while the platelet-to-lymphocyte ratio (PLR) was calculated as platelet count divided by lymphocyte count. The Disease Activity Score for 28 joints (DAS28) was calculated using the following formulas: DAS28-ESR = 0.56 × √(TJC28) + 0.28 × √(SJC28) + 0.70 × ln(ESR) + 0.014 × GH; DAS28-CRP = 0.56 × √(TJC28) + 0.28 × √(SJC28) + 0.36 × ln(CRP + 1) + 0.014 × GH + 0.96 (22). Disease activity was categorized according to the following cutoff values for both methods: high activity (≥5.1), moderate activity (≥3.2 to <5.1), low activity (2.6 to <3.2), and remission (<2.6) (22).

### 2.3 Statistical analysis

For continuous variables, mean ± standard deviation (SD) or median [interquartile range (IQR)] were calculated, and differences were assessed via analysis of variance (ANOVA) or Kruskal–Wallis tests. Categorical variables were presented as numbers (percentages) and evaluated by χ^2^ tests. For continuous variables with a low proportion of missing data (<1%), such as RF, missing data were filled in using the corresponding mean. In cases where the proportion of missing data was high (>10%), as with TNF-α and IL-6, dummy variables indicating missingness were created. Details regarding missing covariates are provided in **Supplementary Table S1**. Additionally, RF, TNF-α, IL-6, ALT, and AST were transformed using the natural logarithm prior to regression analysis.

Simple correlation analyses were carried out with Spearman’s (ρ) correlation coefficients. Multivariable linear regression models were used to assess the association between FAR and DAS28 in four progressive stages: Model 1 was unadjusted; Model 2 included adjustments for age and sex; and Model 3 further adjusted for hypertension, neutrophil count, hemoglobin, platelet count, RF, IL-6, TNF-α, glucocorticoids, methotrexate, leflunomide, cDMARDs, bDMARDs, and tsDMARDs. Model 4 was adjusted for all covariables in Model 3 plus ALT and AST. These confounders were selected on a change in effect of more than 10% or a regression coefficient *p-*value of less than 0.1 (Model 3). To ensure the robustness of our multivariate linear regression models, we conducted a formal assessment for potential multicollinearity among the independent variables. The variance inflation factor (VIF) was calculated for each covariate included in the final models. As detailed in **Supplementary Table S2**, the VIF values for all covariates were well within acceptable limits (all VIFs < 5, with the highest being 2.8 for cDMARDs), confirming the absence of significant multicollinearity. Sensitivity analyses were conducted to assess the robustness of these findings. FAR was categorized according to tertiles, and *p* for trend was determined by considering the median FAR of each tertile as a continuous variable. Potential nonlinear relationships between FAR and DAS28 were investigated using generalized additive models. When nonlinearity was detected, threshold effects on DAS28-CRP and DAS28-ESR were estimated using two-piecewise linear regression. Inflection points were identified through smoothing plots combined with a recursive algorithm to maximize model likelihood. Finally, receiver operating characteristic (ROC) curve analyses were employed to evaluate the effectiveness of FAR in distinguishing high from moderate disease activity. The optimal cutoff point, along with corresponding specificity and sensitivity, was identified by the Youden index.

All data analyses were performed with R software (http://www.r-project.org, The R Foundation) and EmpowerStats (http://www.empowerstats.com, X&Y Solutions, Inc., Boston, MA), with statistical significance defined as a two-sided *p*-value less than 0.05.

## 3 Results

### 3.1 Study population characteristics

Our study included 981 patients with RA (77.88% female), with a mean age of 57.69 ± 12.78 years, and a median disease duration of 48.00 months (IQR: 12.00–132.00). Notably, as all participants were inpatients, the cohort exhibited a characteristic inpatient profile, with a small proportion of patients with low disease activity or remission (DAS28-ESR < 3.2; *n* = 43, 4.4%).

As shown in **Table 1**, when stratified by DAS28-ESR, patients with high disease activity (≥5.1, *n* = 534), compared to those with moderate activity (≥3.2 to <5.1, *n* = 404) and remission/low activity (<3.2, *n* = 43), were more likely to be male and older. They also had significantly higher inflammatory markers (including ESR, CRP, RF, IL-6, WBC counts, neutrophil counts, and platelet counts), but lower RBC counts, hemoglobin, and albumin levels, along with notably elevated fibrinogen and FAR values (all *p* < 0.05). Regarding treatment, patients with high disease activity were less likely to be treated with methotrexate, leflunomide, types of csDMARDs, and tsDMARDs, while bDMARDs were used less often in both the low and high disease activity groups. However, these variables including BMI, smoking status, alcohol use, disease duration, NSAID/glucocorticoid use, lymphocyte counts, ALT, AST, and autoantibody status were similar among the groups.

**Table 1:** Clinical characteristics of patients with RA stratified by DAS28-ESR (*N* = 981).

Characteristics	DAS28ESR				*P-*value
	Total	<3.2	≥3.2, <5.1	≥5.1	
*N* (%)	981 (100%)	43 (4.38%)	404 (41.18%)	534 (54.44%)	
Female, *n* (%)	764 (77.88%)	35 (81.40%)	333 (82.43%)	396 (74.16%)	0.009
Age (years)	57.69 (12.78)	54.23 (13.90)	54.72 (13.01)	60.21 (11.96)	<0.001
BMI (kg/m^2^)	23.99 (3.49)	23.98 (3.44)	24.14 (3.57)	23.87 (3.44)	0.497
Smoking, *n* (%)	55 (5.61%)	4 (9.30%)	17 (4.21%)	34 (6.37%)	0.203
Drinking, *n* (%)	18 (1.83%)	1 (2.33%)	7 (1.73%)	10 (1.87%)	0.958
HP, *n* (%)	321 (32.72%)	13 (30.23%)	114 (28.22%)	194 (36.33%)	0.030
DM, *n* (%)	91 (9.28%)	7 (16.28%)	26 (6.44%)	58 (10.86%)	0.019
CHD, *n* (%)	68 (6.93%)	2 (4.65%)	32 (7.92%)	34 (6.37%)	0.542
DD (months)	48 (12–132)	58 (24–138)	57 (12–141)	48 (9–132)	0.328
NSAIDs, *n* (%)	505 (51.48%)	20 (46.51%)	215 (53.22%)	270 (50.56%)	0.579
GLU, *n* (%)	107 (10.91%)	8 (18.60%)	45 (11.14%)	54 (10.11%)	0.224
MTX, *n* (%)	204 (20.80%)	12 (27.91%)	116 (28.71%)	76 (14.23%)	<0.001
LEF, *n* (%)	193 (19.67%)	18 (41.86%)	79 (19.55%)	96 (17.98%)	<0.001
Types of cDMARDs, *n* (%)					<0.001
0	505 (51.48%)	11 (25.58%)	176 (43.56%)	318 (59.55%)	
1	356 (36.29%)	18 (41.86%)	169 (41.83%)	169 (31.65%)	
2	114 (11.62%)	14 (32.56%)	55 (13.61%)	45 (8.43%)	
3	6 (0.61%)	0 (0.00%)	4 (0.99%)	2 (0.37%)	
bDMARDs, *n* (%)	42 (4.28%)	0 (0.00%)	28 (6.93%)	14 (2.62%)	0.002
tsDMARDs, *n* (%)	23 (2.34%)	2 (4.65%)	16 (3.96%)	5 (0.94%)	0.006
WBC (×10^9^/L)	6.53 (2.19)	5.65 (2.05)	6.05 (2.02)	6.96 (2.22)	<0.001
Neut (×10^9^/L)	4.23 (1.81)	3.44 (1.66)	3.78 (1.66)	4.64 (1.84)	<0.001
Lymph (×10^9^/L)	1.63 (0.59)	1.61 (0.55)	1.65 (0.59)	1.62 (0.59)	0.787
RBC (×10^12^/L)	3.88 (0.49)	4.02 (0.63)	3.97 (0.48)	3.80 (0.48)	<0.001
Hb (g/L)	111.08 (17.07)	119.77 (18.55)	114.10 (17.21)	108.11 (16.22)	<0.001
PLT (×10^9^/L)	291.71 (90.96)	238.53 (54.48)	268.15 (85.14)	313.82 (91.43)	<0.001
ALT(U/L)	15.90 (11.20–23.70)	15.10 (11.45–24.45)	16.20 (10.90–25.07)	15.55 (11.20–21.67)	0.369
AST(U/L)	18.10 (14.70–23.80)	18.70 (15.95–24.10)	18.70 (14.90–25.02)	17.90 (14.22–22.82)	0.470
ESR (mm/h)	56.00 (33.00–86.00)	15.00 (9.50–28.00)	32.00 (23.00–50.00)	82.00 (61.00–103.00)	<0.001
CRP (mg/L)	26.01 (7.07–48.16)	3.63 (1.60–8.41)	8.50 (2.89–24.39)	40.93 (24.68–65.51)	<0.001
RF (IU/mL)	161.80 (47.77–338.20)	61.50 (25.65–171.10)	119.95 (37.68–279.40)	206.60 (59.50–427.40)	<0.001
IL-6, pg/mL	38.70 (13.36–86.10)	9.19 (4.51–23.36)	20.04 (7.47–42.99)	61.02 (30.58–114.82)	<0.001
TNF-α, pg/mL	10.00 (2.93–26.17)	9.94 (4.09–25.84)	9.35 (2.76–23.08)	10.37 (2.95–29.08)	0.401
ACPA positive, *n* (%)	883 (90.84%)	37 (88.10%)	362 (90.73%)	484 (91.15%)	0.800
APF positive, *n* (%)	769 (81.03%)	36 (85.71%)	304 (79.17%)	429 (82.03%)	0.406
AKA positive, *n* (%)	710 (74.89%)	31 (73.81%)	279 (72.85%)	400 (76.48%)	0.453
Albumin (g/L)	36.99 (4.83)	39.47 (4.20)	38.47 (4.13)	35.67 (4.97)	<0.001
Fibrinogen (g/L)	4.01 (1.01)	3.11 (0.84)	3.56 (0.84)	4.43 (0.94)	<0.001
FAR	0.11(0.04)	0.08 (0.02)	0.09 (0.03)	0.13 (0.03)	<0.001

Values are presented as mean (SD), median (Q1–Q3) or *n* (%). DAS28ESR, Disease Activity Score 28 using erythrocyte sedimentation rate; BMI, body mass index; HP, hypertension; DM, diabetes mellitus; CHD, coronary heart disease, DD, disease duration; NSAIDs, non-steroidal anti-inflammatory drugs; GLU, glucocorticoids; MTX, methotrexate; LEF, leflunomide; cDMARDs, conventional disease-modifying antirheumatic drugs; bDMARDs, biological disease-modifying antirheumatic drugs; tsDMARDs, targeted synthetic disease-modifying antirheumatic drugs; WBC, white blood cell count; Neut, neutrophil count; Lymph, lymphocyte count; RBC, red blood cell; HB, hemoglobin; PLT, platelet; ALT, alanine aminotransferase; AST, aspartate aminotransferase; ESR, erythrocyte sedimentation rate; CRP, C-reactive protein; RF, rheumatoid factor; TNF-α, tumor necrosis factor α; IL-6, interleukin-6; ACPA, anti-citrullinated protein antibody; AKA, anti-keratin antibody; APF, anti-perinuclear factor; FAR, fibrinogen-to-albumin ratio.

As shown in **Supplementary Table S3**, analysis by FAR tertiles revealed that patients in the highest tertile (T3) shared similar characteristics to the high disease activity group: male predominance, older age, elevated inflammatory markers, and reduced RBC counts, hemoglobin, and albumin levels. These patients were less frequently prescribed methotrexate and types of csDMARDs but had higher glucocorticoid use (*p* < 0.05). No meaningful variations were detected among FAR tertiles in terms of BMI, smoking or alcohol habits, disease duration, lymphocyte counts, or ACPA/AKA/APF positivity.

### 3.2 Univariate analysis

As summarized in **Supplementary Table S4**, univariate analysis indicated that male sex, older age, and hypertension were significantly associated with higher disease activity. Increased disease activity also correlated with elevated inflammatory markers, including WBC count, neutrophil count, RF, IL-6, platelet count, fibrinogen, and FAR. In contrast, higher RBC count, hemoglobin, albumin levels, and AST were linked to lower disease activity. Use of csDMARDs (methotrexate and leflunomide) and tsDMARDs was negatively associated with disease activity. No significant associations were found for BMI, alcohol use, CHD, lymphocyte count, ALT, and the use of NSAIDs, glucocorticoids, and bDMARDs.

### 3.3 Correlation analysis of FAR and its components with traditional inflammatory markers

As shown in **Supplementary Figure S1**, Spearman correlation analysis revealed that FAR was positively correlated with both ESR (ρ = 0.70, *p* < 0.001) and CRP (ρ = 0.72, *p* < 0.001). Notably, these correlations were stronger than those observed for the individual components of FAR. Specifically, fibrinogen showed moderate positive correlations with ESR and CRP (ρ = 0.64 and 0.65, respectively), whereas albumin exhibited weak negative correlations (ρ = −0.40 and −0.45, respectively).

### 3.4 Association of FAR with disease activity in rheumatoid arthritis

As shown in **Table 2**, multiple linear regression analysis demonstrated a robust positive relationship between FAR and disease activity across all models. In the fully adjusted Model 4, each unit increase in FAR corresponded to an 11.18-point elevation in DAS28-ESR [95% confidence interval (CI): 9.55–12.81] and a 10.07-point increase in DAS28-CRP (95% CI: 8.59–11.54). Supporting these findings, sensitivity analysis using FAR tertiles revealed that patients in the highest tertile (T3) exhibited significantly higher disease activity compared to those in the lowest tertile (T1), with increased DAS28-ESR (β = 0.94, 95% CI: 0.80–1.08) and DAS28-CRP (β = 0.83, 95% CI: 0.70–0.96). This association persisted consistently throughout all models.

**Table 2:** Multivariate linear regression results of association between FAR and DAS28.

Exposure	Model 1 β (95% CI)	Model 2 β (95% CI)	Model 3 β (95% CI)	Model 4 β (95% CI)
DSA28ESR				
FAR	15.48 (14.08, 16.87)	15.17 (13.70, 16.64)	11.17 (9.55, 12.79)	11.18 (9.55, 12.81)
FAR (tertiles)				
T1	Ref	Ref	Ref	Ref
T2	0.70 (0.58, 0.83)	0.67 (0.55, 0.80)	0.52 (0.40, 0.64)	0.52 (0.40, 0.64)
T3	1.34 (1.21, 1.46)	1.30 (1.17, 1.43)	0.94 (0.80, 1.07)	0.94 (0.80, 1.08)
*p* for trend	<0.0001	<0.0001	<0.0001	<0.0001
DAS28CRP				
FAR	15.28 (13.96, 16.60)	14.70 (13.30, 16.09)	10.07 (8.60, 11.53)	10.07 (8.59, 11.54)
FAR (tertiles)				
T1	Ref	Ref	Ref	Ref
T2	0.69 (0.57, 0.81)	0.66 (0.54, 0.78)	0.47 (0.36, 0.58)	0.47 (0.36, 0.58)
T3	1.31 (1.19, 1.43)	1.25 (1.13, 1.37)	0.83 (0.70, 0.95)	0.83 (0.70, 0.96)
*p* for trend	<0.0001	<0.0001	<0.0001	<0.0001

Model 1 was adjusted for none; Model 2 was adjusted for sex and age; Model 3 was adjusted for sex, age, hypertension, neutrophil count, hemoglobin, platelet count, rheumatoid factor, interleukin-6, tumor necrosis factor α, glucocorticoids, methotrexate, leflunomide, conventional disease-modifying antirheumatic drugs, targeted synthetic disease-modifying antirheumatic drugs, and biological disease-modifying antirheumatic drugs. Model 4 was adjusted for all covariables in Model 3 plus alanine aminotransferase and aspartate aminotransferase.

### 3.5 Nonlinear relationship of FAR with disease activity in RA

**Figure 2:**
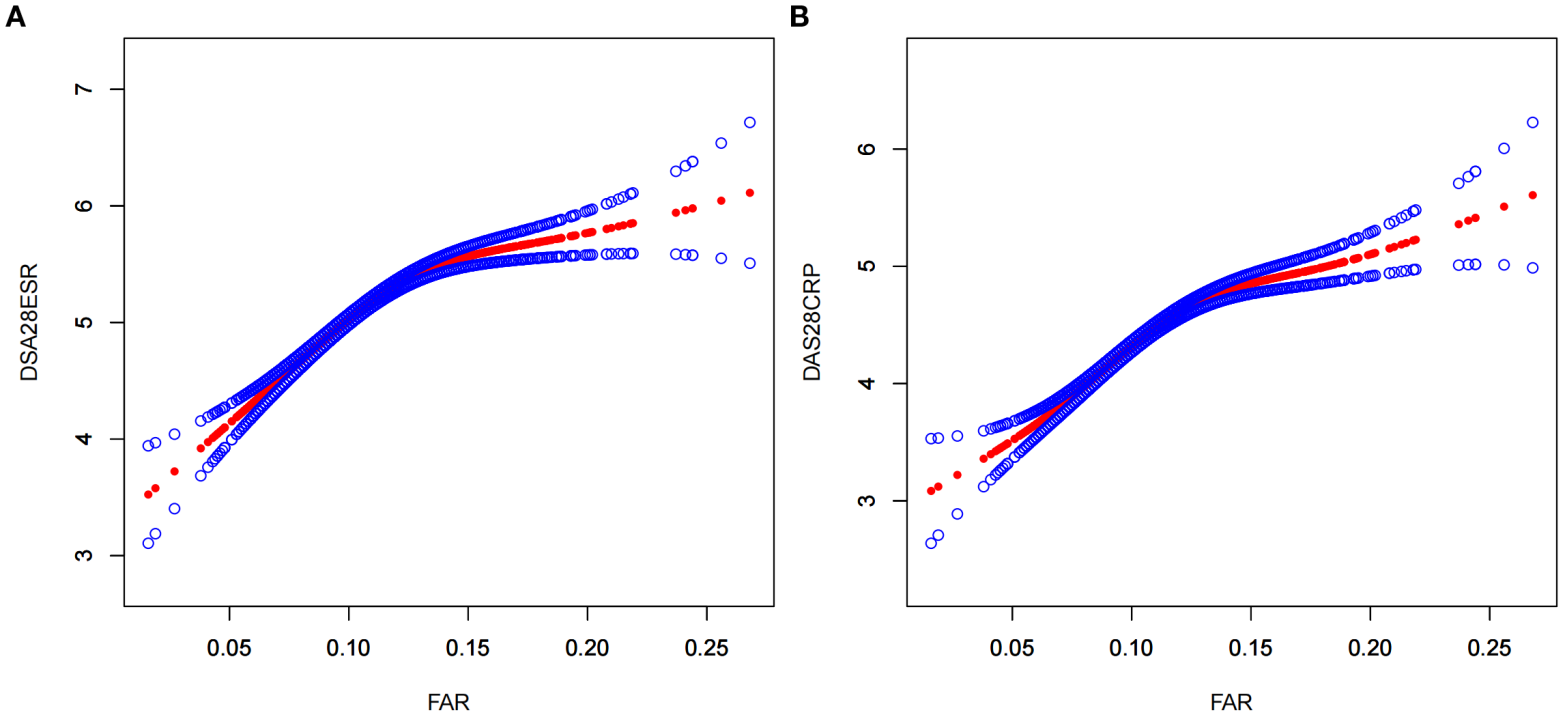
A nonlinear relationship of FAR with DAS28-ESR **(A)** and DAS28-ESR **(B)**. The models were adjusted for sex, age, hypertension, neutrophil count, hemoglobin, platelet count, alanine aminotransferase, aspartate aminotransferase; rheumatoid factor, interleukin-6, tumor necrosis factor α, glucocorticoids, methotrexate, leflunomide, conventional disease-modifying antirheumatic drugs, biological disease-modifying antirheumatic drugs, and targeted synthetic disease-modifying antirheumatic drugs. FAR, fibrinogen-to-albumin ratio; DAS28ESR, Disease Activity Score 28 using erythrocyte sedimentation rate; DAS28CRP, Disease Activity Score 28 using C-reactive protein.

As shown in **Figure 2**, a nonlinear relationship of FAR with disease activity was observed after adjusting for numerous confounding factors. As shown in **Table 3**, a clear threshold effect was identified at FAR = 0.14 for both DAS28-CRP and DAS28-ESR. When FAR was below this threshold, each unit increase was strongly positively associated with disease activity (DAS28-ESR: β = 15.21, 95% CI: 13.01–17.42; DAS28-CRP: β = 13.07, 95% CI: 11.07–15.07). Conversely, when FAR exceeded 0.14, this association was markedly attenuated and no longer statistically significant (DAS28-ESR: β = 2.19, 95% CI: −1.53–5.92; DAS28-CRP: β = 3.37, 95% CI: −0.01–6.74).

**Table 3:** The result of the two-piecewise linear regression model of FAR with DAS28-ESR and DAS28-CRP.

	DAS28-ESR β (95% CI)	DAS28-CRP β (95% CI)
Fitting model by standard linear regression	11.18 (9.55, 12.81)	10.07 (8.59, 11.54)
Fitting model by two-piecewise linear regression		
Inflection point of FAR	0.14	0.14
<0.14	15.21 (13.01, 17.42)	13.07 (11.07, 15.07)
>0.14	2.19 (−1.53, 5.92)	3.37 (−0.01, 6.74)
*p* for log likelihood ratio test	<0.001	<0.001

The models were adjusted for sex, age, hypertension, neutrophil count, hemoglobin, platelet count, alanine aminotransferase, aspartate aminotransferase, rheumatoid factor, interleukin-6, tumor necrosis factor α, glucocorticoids, methotrexate, leflunomide, conventional disease-modifying antirheumatic drugs, biological disease-modifying antirheumatic drugs, and targeted synthetic disease-modifying antirheumatic drug. PAR, platelet-to-albumin ratio; DAS28ESR, Disease Activity Score 28 using erythrocyte sedimentation rate; DAS28CRP, Disease Activity Score 28 using C-reactive protein.

### 3.6 Evaluation of the discriminatory power of FAR for high disease activity

**Figure 3:**
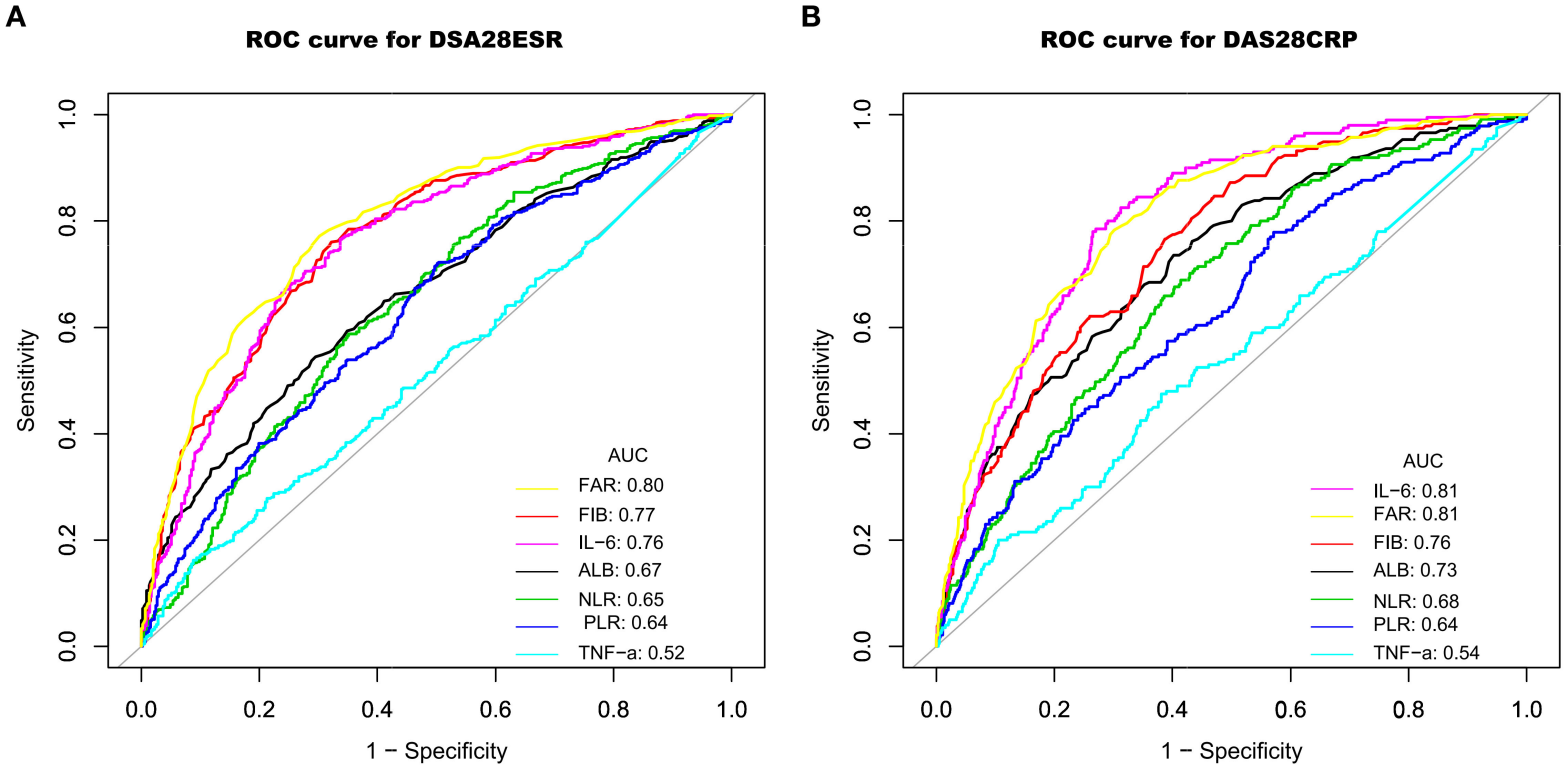
ROC curves demonstrating the ability to identify high disease activity using DAS28-ESR **(A)** and DAS28-CRP **(B)**, based on the biomarkers FIB, ALB, NLR, PLR, IL-6, TNF-α, and FAR. ROC, receiver operator characteristic; AUC, area under the curve; FIB, fibrinogen; ALB, albumin; TNF-α, tumor necrosis factor α; IL-6, interleukin-6; NLR, neutrophil-to-lymphocyte ratio; PLR, platelet-to-lymphocyte ratio; FAR, fibrinogen-to-albumin ratio; DAS28ESR, Disease Activity Score 28 using erythrocyte sedimentation rate; DAS28CRP, Disease Activity Score 28 using C-reactive protein.

The ability of FAR to distinguish high from moderate disease activity was evaluated using ROC curve analysis. As illustrated in **Figure 3** and detailed in **Table 4**, FAR exhibited comparable or better discriminatory power compared with other inflammatory markers, including fibrinogen, albumin, NLR, PLR, IL-6, and TNF-α. Specifically, using the DAS28-ESR and DAS28-CRP criteria, FAR achieved an area under the curve (AUC) of 0.80 (95% CI: 0.77–0.82) and 0.81 (95% CI: 0.78–0.84), respectively. These AUCs were higher than those for fibrinogen (0.77 and 0.76), albumin (0.67 and 0.73), NLR (0.65 and 0.68), PLR (0.64 and 0.64), and TNF-α (0.52 and 0.54). Notably, the performance of FAR was comparable to, or slightly better than, that of IL-6 (AUCs: 0.76 and 0.81).

**Table 4:** AUCs of inflammatory markers in predicting high disease activity.

Variables	AUC (95% CI)	Sensitivity	Specificity	Youden index	Cutoff
DAS28ESR					
Fibrinogen	0.77 (0.74, 0.80)	0.76	0.68	0.44	3.81
Albumin	0.67 (0.63, 0.70)	0.54	0.70	0.24	36.45
FAR	0.80 (0.77, 0.82)	0.77	0.70	0.47	0.10
NLR	0.65 (0.61,0.68)	0.59	0.65	0.24	2.66
PLR	0.64 (0.60, 0.67)	0.72	0.50	0.22	159.58
IL-6	0.76 (0.73, 0.80)	0.68	0.75	0.43	39.80
TNF-α	0.52 (0.48, 0.56)	0.16	0.91	0.07	54.73
DAS28CRP					
Fibrinogen	0.76 (0.72, 0.79)	0.77	0.61	0.38	4.03
Albumin	0.73 (0.69, 0.76)	0.74	0.60	0.34	37.05
FAR	0.81 (0.78, 0.84)	0.78	0.70	0.48	0.11
NLR	0.68 (0.64, 0.72)	0.69	0.59	0.28	2.66
PLR	0.64 (0.60, 0.68)	0.78	0.43	0.21	159.58
IL-6	0.81 (0.78, 0.84)	0.78	0.73	0.51	52.16
TNF-α	0.54 (0.49, 0.59)	0.20	0.89	0.19	54.73

AUC, area under the curve; FAR, fibrinogen-to-albumin ratio; NLR, neutrophil-to-lymphocyte ratio, PLR, platelet-to-lymphocyte ratio; IL-6, interleukin-6; TNF-α, tumor necrosis factor α; DAS28ESR, Disease Activity Score 28 using erythrocyte sedimentation rate; DAS28CRP, Disease Activity Score 28 using C-reactive protein.

## 4 Discussion

In this cross-sectional study of 981 hospitalized patients with RA, predominantly with moderate to high disease activity, we identified a nonlinear relationship between FAR and disease activity, as assessed by both DAS28-ESR and DAS28-CRP, independent of potential confounders. A saturation threshold effect was found at a FAR value of 0.14. Below this cutoff, FAR exhibited a positive association with both DAS28-CRP and DAS28-ESR, whereas no significant association was observed at higher FAR levels. Additionally, compared to other markers such as NLR, PLR, TNF-α, and IL-6, FAR demonstrated comparable or better discriminatory power for detecting high from moderate disease activity, with AUCs of 0.80 (95% CI: 0.77–0.82) for DAS28-ESR and 0.81 (95% CI: 0.78–0.84) for DAS28-CRP.

The clinical utility of an inflammatory marker is defined not only by its underlying biological relationship with a disease state but also by its performance as a diagnostic tool. Our findings reveal that these two aspects are synergistic for FAR. Although we identified a saturation threshold at FAR = 0.14, beyond which its association with DAS28 plateaus, this does not diminish its value in distinguishing high from moderate disease activity. Instead, this threshold defines the optimal operational range for its clinical application. Our ROC analysis of the entire cohort identified optimal cutoffs of 0.10 for DAS28-ESR and 0.11 for DAS28-CRP. Crucially, these diagnostic thresholds fall below the saturation point, precisely within the range where FAR demonstrates its strongest and most linear association with disease activity. This alignment confirms that the maximal discriminatory power of FAR is achieved by leveraging its performance in this sensitive, sub-threshold range. Therefore, the threshold analysis elucidates the biological boundaries of FAR as a dynamic marker, while the ROC analysis translates this understanding into a practical, single-value tool for clinical decision-making, confirming its utility in identifying high-risk patients who may warrant more aggressive therapeutic strategies.

RA involves complex inflammatory processes, and reliable biomarkers are crucial for effective disease management (23). While traditional inflammatory markers such as CRP and ESR are widely used, they have notable limitations in sensitivity and specificity (7). Recent studies indicate that FAR may serve as a valuable marker in various autoimmune and inflammatory conditions. For example, Ding et al. reported significantly elevated FAR levels in patients with spondyloarthritis compared to those with osteoarthritis and healthy controls, with the highest levels observed in reactive arthritis (17). Similarly, Liu et al. found that FAR was markedly increased in patients with AS and showed a positive association with Bath Ankylosing Spondylitis Disease Activity Index scores (*r* = 0.594, *p* < 0.001), demonstrating high diagnostic accuracy for assessing disease activity (16). Furthermore, Dai et al. identified FAR as a significant predictor of disease activity, including active, severe, and poor prognosis states in SLE, with positive correlations to the SLE Disease Activity Index, highlighting its potential as a biomarker for disease assessment and prognosis (15). Although some studies have explored the albumin-to-fibrinogen ratio (AFR, the inverse of FAR) in RA and reported associations with ESR, CRP, RF, DAS28, and Th17 cell ratios (24–26), these investigations were primarily limited to basic correlation analyses with small sample sizes. In contrast, the present study employed advanced statistical techniques to, for the first time, identify a nonlinear relationship between FAR and RA disease activity. Specifically, we revealed a threshold effect at FAR = 0.14, FAR exhibited a positive association with both DAS28-CRP and DAS28-ESR only in patients with FAR < 0.14, whereas no significant association was observed at higher FAR levels.

The observed non-linear relationship between FAR and disease activity, particularly the saturation effect at the 0.14 threshold, warrants deeper mechanistic exploration. While methodological factors such as sample size or data variability could theoretically contribute to this phenomenon, our supplementary analysis confirms that the subgroup above the threshold (*n* = 159) remains substantial and the data distribution is robust (**Supplementary Figures S2**, **S3**). This suggests that the attenuation of the association is not merely a statistical artifact. We therefore postulate that this phenomenon is underpinned by complex homeostatic regulatory mechanisms. For instance, as the inflammatory response intensifies, the body may activate negative feedback pathways to preserve homeostasis; high levels of pro-inflammatory signals, for example, could concurrently induce anti-inflammatory mediators like IL-10, thereby tempering further fibrinogen synthesis. Furthermore, the liver, the primary site of synthesis, might undergo functional adaptation—either reaching its production capacity or actively downregulating acute-phase protein synthesis to prevent metabolic overload under sustained, intense stimulation. Finally, in advanced disease stages, the DAS28 score may increasingly reflect persistent symptoms attributable to structural damage, whereas FAR primarily marks the intensity of current inflammation, leading to a potential decoupling of the two metrics. We therefore hypothesize that the plateau phase observed at FAR > 0.14 signifies a transition from a state of straightforward inflammation to one where active homeostatic counter-regulation is engaged, although this hypothesis requires further validation through basic research.

In the assessment of inflammation in RA, fibrinogen serves as a valuable supplementary biomarker that bridges ESR and CRP. Clinical data indicate that ESR and CRP measurements can show discordance in approximately 25%–30% of patients with RA (22, 27). Fibrinogen has intermediate temporal kinetics, typically peaking within 3–5 days of inflammatory stimulation and maintaining a similar half-life (28). This unique temporal pattern allows fibrinogen levels to remain elevated for up to 6 weeks during inflammation resolution, often after CRP has normalized. Such prolonged elevation enhances the detection of subclinical inflammation. FAR further enhances this benefit by combining the rapid decline of albumin with the slower normalization of fibrinogen. Our findings further confirm that FAR exhibits stronger positive correlations with both ESR (ρ = 0.70) and CRP (ρ = 0.72) than fibrinogen or albumin alone. Moreover, FAR exhibits significant discriminatory accuracy in distinguishing high from moderate disease activity compared to its individual components. Specifically, FAR achieved AUC values of 0.80 (DAS28-ESR) and 0.81 (DAS28-CRP), significantly outperforming fibrinogen (0.77 and 0.76) and albumin (0.67 and 0.73). These findings suggest that FAR is not simply a composite of its two components, but rather provides a more comprehensive and balanced assessment of inflammation.

As an acute-phase protein, fibrinogen is primarily synthesized and secreted by the liver during inflammatory responses, a process largely mediated by IL-6 (29–31). Fibrinogen can directly regulate leukocyte functions by binding to β2 integrin receptors—including αMβ2 and αXβ2—on the surface of leukocytes. This interaction promotes leukocyte migration, phagocytosis, the secretion of pro-inflammatory mediators, and the activation of NF-κB signaling (32–38). Additionally, fibrinogen induces the production of IL-1β and TNF-α in monocytes and stimulates the expression of chemokines such as IL-8 and MCP-1 in endothelial cells and fibroblasts (30, 31, 39). In RA, fibrin deposits in synovial tissues and joint cavities facilitate leukocyte infiltration, and disrupting the fibrinogen–αMβ2 interaction can attenuate arthritis progression without impairing coagulation (40, 41). Notably, fibrinogen can also activate platelets, leading to the release of IL-1β and CD40L (42), and may promote osteoclast differentiation through the RANK/RANKL pathway (40, 43). Although preclinical evidence suggests that fibrinogen can promote osteoclast differentiation and bone erosion via the RANK/RANKL pathway, our current study, lacking imaging data on bone erosion, cannot directly validate the clinical association between FAR and erosive disease in patients with RA. This represents a significant limitation and highlights a crucial direction for future investigation. We propose that prospective, longitudinal studies, integrating serial FAR measurements with quantitative imaging assessments of bone erosion progression, are warranted to rigorously evaluate the potential of FAR as a predictive biomarker for joint damage.

This study has several notable strengths. Firstly, this study included 981 hospitalized patients with RA, providing a sufficiently large sample size that offers substantial statistical power to robustly investigate the relationship between FAR and disease activity. Secondly, by utilizing both DAS28-ESR and DAS28-CRP, the study delivers a comprehensive and objective assessment of RA activity, thereby enhancing the robustness of the findings. Thirdly, we employed rigorous multivariate regression analyses to meticulously control for a wide array of potential confounders to effectively minimize bias. These included demographic factors (gender and age), a broad spectrum of RA medications (glucocorticoids, csDMARDs, bDMARDs, and tsDMARDs), and other key inflammatory markers (neutrophil count, platelet count, RF, IL-6, and TNF-α). Furthermore, we specifically addressed the potential influence of hepatic function, a critical consideration given the liver’s role in synthesizing both fibrinogen and albumin and the known hepatotoxic potential of certain DMARDs. Our analysis revealed that liver enzyme levels neither correlated with disease activity nor attenuated the strong, independent link between FAR and DAS28, confirming that our findings are not confounded by liver function. Most importantly, this study is the first to reveal a nonlinear relationship between FAR and DAS28, with a saturation threshold effect, and has demonstrated its significant discriminatory performance in distinguishing high disease activity from moderate disease activity. This key finding offers clinicians a simple, practical tool to supplement the existing disease activity assessment systems.

However, several limitations should be acknowledged. First, our study was confined to hospitalized patients with RA, which resulted in a limited sample size for the low disease activity/remission subgroup. Consequently, our conclusions are primarily applicable to patients with moderate to high disease activity. Further validation in cohorts with a higher representation of outpatients or patients in stable remission is warranted to assess the utility of FAR across the full disease activity spectrum. Second, as a cross-sectional study, it can only establish associations at a single time point and does not track the dynamic changes in FAR and DAS28 in response to treatment. Third, although the study carefully adjusted for multiple confounders, the potential impact of unmeasured residual confounding cannot be entirely excluded. Finally, although we collected data on pre-existing cardiovascular conditions (e.g., CHD and hypertension), the number of affected cases was insufficient to conduct a reliable subgroup analysis. Consequently, we were unable to assess the potential of FAR to predict cardiovascular risk in patients with RA, despite its established status as a recognized CVD risk marker in the general population. Therefore, multicenter prospective cohort studies are needed to further validate these findings.

In summary, the present study reveals a nonlinear association between FAR and RA disease activity, characterized by a saturation threshold effect. A strong positive association was observed between FAR and both DAS28-ESR and DAS28-CRP when FAR values were below 0.14; however, this correlation diminished and was no longer evident at higher FAR levels. In addition, FAR exhibits comparable or better discriminatory performance in distinguishing high disease activity from moderate disease activity compared with other inflammatory markers, including IL-6, TNF-α, NLR, and PLR. These findings suggest that FAR may serve as a simple, readily accessible, and cost-effective inflammatory marker to supplement existing disease activity assessment tools, helping to rapidly identify high-risk patients. Nevertheless, these conclusions are mainly derived from hospitalized patients with RA with moderate to high disease activity at a single center. Validation studies across more diverse populations and multicenter settings are warranted.

## Data Availability

The original contributions presented in the study are included in the article/**Supplementary Material**. Further inquiries can be directed to the corresponding author.
