# Supplementary material for: The potential value of fibrinogen-to-albumin ratio in assessing disease activity in rheumatoid arthritis
Source: Front Immunol. 2025 Nov 18;16:1670731. doi: 10.3389/fimmu.2025.1670731 (PMC12669121; doi:10.3389/fimmu.2025.1670731)

Supplementary Material

Supplementary Tables

Supplementary Table S1. Description of the missing variables

Variables	No.	Missing data, N
N	981	0
Female	981	0
Age	981	0
BMI	976	5
Smoking	981	0
Drinking	981	0
Hp	981	0
DM	981	0
CHD	981	0
DD	981	0
SJC28	981	0
TJC28	981	0
VAS	981	0
ESR	981	0
CRP	981	0
Albumin	981	0

RF	972	9
IL-6	792	189
TNF-a	790	191
Fibrinogen	981	0
WBC,	981	0
Neut,	981	0
Lymph	981	0
RBC	981	0
Hb	981	0
PLT	981	0
ALT	981	0
AST	981	0
CCP	972	9
AKA	949	32
APF	948	33
NSAIDs	981	0
Glu	981	0
MTX	981	0
LEF	981	0
cDMARDs	981	0
tsDMARDs	981	0

bDMARDs	981	0

BMI, body mass index; HP, Hypertension; DM, Diabetes Mellitus; CHD, Coronary Heart Disease, DD, disease duration; SJC28, swollen joint count in 28 joints; TJC28, tender joint count in 28 joints; VAS, visual analogue scale; ESR, Erythrocyte sedimentation rate; CRP, C-reactive protein; RF, rheumatoid factor; TNF-α, tumor necrosis factor α; IL-6, interleukin-6; WBC, white blood cell count; Neut, neutrophil count; Lymph, lymphocyte count; RBC, red blood cell; HB, Hemoglobin; PLT, platelet; ALT, alanine aminotransferase, aspartate aminotransferase; ACPA, anti-citrullinated protein antibody; AKA, anti-keratin antibody; APF, anti-perinuclear factor; NSAIDs, non-steroidal anti-inflammatory drugs; GLU, Glucocorticoids; MTX, Methotrexate; LEF, Leflunomide; cDMARDs, conventional disease-modifying antirheumatic drugs; bDMARDs, biological disease-modifying antirheumatic drugs; tsDMARDs, targeted synthetic disease-modifying antirheumatic drugs.

Supplementary Table S2. A comprehensive multicollinearity assessment on all covariates included in the final adjusted models

Variables	Variance Inflation Factor
Sex	1.2
Age	1.3
Hypertension	1.2
Neutrophil Count	1.4
Hemoglobin	1.4
Platelet Count	1.3
Rheumatoid Factor	1.1
Interleukin-6	1.9
Tumor necrosis factor α	1.8
Fibrinogen to albumin ratio	1.4
Alanine aminotransferase	2.1
Aspartate aminotransferase	2.1
Glucocorticoids	1.1
Methotrexate	2.0
Leflunomide	1.9
Conventional disease-modifying antirheumatic drugs	2.8
Biological disease-modifying antirheumatic drugs	1.0
Targeted synthetic disease-modifying antirheumatic drugs	1.0

Supplementary Table S3. Clinical characteristics of RA patients according to tertiles of FAR

Characteristics	Total	Tertiles of FAR			*P* value
		T1 0.02-0.09	T2 0.09-0.12	T3 0.12-0.27	
N	981	319	334	328	
Female, n (%)	764 (77.88%)	274 (85.89%)	268 (80.24%)	222 (67.68%)	<0.001
Age, year	57.69 (12.78)	52.62 (13.13)	58.35 (12.10)	61.94 (11.36)	<0.001
BMI, kg/m^2	23.99 (3.49)	24.06 (3.60)	24.12 (3.42)	23.78 (3.46)	0.415
Smoking, n (%)	55 (5.61%)	13 (4.08%)	18 (5.39%)	24 (7.32%)	0.196
Drinking, n (%)	18 (1.83%)	4 (1.25%)	6 (1.80%)	8 (2.44%)	0.531
Hp, n (%)	321 (32.72%)	79 (24.76%)	114 (34.13%)	128 (39.02%)	<0.001
DM, n(%)	91 (9.28%)	20 (6.27%)	40 (11.98%)	31 (9.45%)	0.042
CHD, n(%)	68 (6.93%)	14 (4.39%)	29 (8.68%)	25 (7.62%)	0.081
DD, month	48.00 (12.00-132.00)	48.00 (10.50-120.00)	60.00 (12.00-136.50)	52.00 (12.00-144.00)	0.244
NSAIDs, n (%)	505 (51.48%)	177 (55.49%)	178 (53.29%)	150 (45.73%)	0.033
Glu, n (%)	107 (10.91%)	31 (9.72%)	28 (8.38%)	48 (14.63%)	0.025
MTX, n (%)	204 (20.80%)	90 (28.21%)	82 (24.55%)	32 (9.76%)	<0.001
LEF, n (%)	193 (19.67%)	55 (17.24%)	67 (20.06%)	71 (21.65%)	0.362
Types of cDMARDs, n (%)					0.012
0	505 (51.48%)	152 (47.65%)	160 (47.90%)	193 (58.84%)	
1	356 (36.29%)	124 (38.87%)	123 (36.83%)	109 (33.23%)	
2	114 (11.62%)	42 (13.17%)	47 (14.07%)	25 (7.62%)	

3	6 (0.61%)	1 (0.31%)	4 (1.20%)	1 (0.30%)	
tsDMARDs, n (%)	23 (2.34%)	17 (5.33%)	2 (0.60%)	4 (1.22%)	<0.001
bDMARDs, n (%)	42 (4.28%)	13 (4.08%)	19 (5.69%)	10 (3.05%)	0.239
WBC, 10^9/L	6.53 (2.19)	5.94 (2.09)	6.47 (2.03)	7.16 (2.27)	<0.001
Neut, 10^9/L	4.23 (1.81)	3.66 (1.65)	4.18 (1.65)	4.85 (1.93)	<0.001
Lymph, 10^9/L	1.63 (0.59)	1.70 (0.63)	1.63 (0.58)	1.56 (0.55)	0.022
RBC, 10^{12}/L	3.88 (0.49)	4.01 (0.49)	3.88 (0.48)	3.76 (0.48)	<0.001
Hb, g/L	111.08 (17.07)	115.05 (17.59)	112.75 (15.78)	105.54 (16.42)	<0.001
PLT, 10^9/L	291.71 (90.96)	264.19 (74.41)	282.52 (88.23)	327.83 (96.71)	<0.001
ALT(U/L)	15.90 (11.20-23.70)	17.30 (12.10-26.15)	15.40 (11.12-22.60)	15.45 (10.28-22.15)	0.005
AST(U/L)	18.10 (14.70-23.80)	19.70 (15.75-25.60)	17.90 (14.70-23.80)	17.25 (13.80-22.02)	<0.001
Fibrinogen, g/L	4.01 (1.01)	3.00 (0.52)	4.00 (0.44)	5.02 (0.75)	<0.001
Albumin, g/L	36.99 (4.83)	40.09 (3.98)	37.57 (3.60)	33.38 (4.29)	<0.001
ESR, mm/H	56.00 (33.00-86.00)	29.00 (19.00-45.00)	54.00 (38.00-76.00)	90.00 (71.00-109.00)	<0.001
CRP, mg/L	26.01 (7.07-48.16)	4.96 (1.72-14.70)	24.28 (9.08-39.74)	50.70 (35.71-79.54)	<0.001
RF, IU/mL	161.80 (47.77-338.20)	137.80 (42.80-298.30)	162.90 (48.00-326.98)	184.70 (51.20-416.70)	0.029
IL-6, pg/mL	38.70 (13.36-86.10)	15.61 (5.45-40.30)	33.65 (13.75-78.58)	74.72 (43.71-131.25)	<0.001
TNF-α, pg/mL	10.00 (2.93-26.17)	8.39 (2.50-21.29)	10.91 (3.99-29.41)	9.79 (3.04-29.38)	0.029
ACPA positive n (%)	883 (90.84%)	289 (91.17%)	307 (93.60%)	287 (87.77%)	0.034
APF positive, n (%)	710 (74.89%)	244 (80.26%)	262 (81.62%)	263 (81.17%)	0.908
AKA positive, n (%)	769 (81.03%)	224 (73.68%)	245 (76.56%)	241 (74.38%)	0.685

DSA28ESR	5.09 (0.97)	4.40 (0.89)	5.11 (0.83)	5.74 (0.70)	<0.001
DAS28CRP	4.39 (0.93)	3.72 (0.82)	4.41 (0.77)	5.03 (0.71)	<0.001

Values are presented as mean (SD), median (Q1–Q3) or n (%). FAR, fibrinogen-to-albumin ratio; BMI, body mass index; HP, Hypertension; DM, Diabetes Mellitus; CHD, Coronary Heart Disease, DD, disease duration; NSAIDs, Non-Steroidal Anti-Inflammatory Drugs; GLU, Glucocorticoids; MTX, Methotrexate; LEF, Leflunomide; cDMARDs, conventional disease-modifying antirheumatic drugs; tsDMARDs, targeted synthetic disease-modifying antirheumatic drugs; bDMARDs, biological disease-modifying antirheumatic drugs; WBC, white blood cell count; Neut, neutrophil count; Lymph, lymphocyte count; RBC, red blood cell; HB, hemoglobin; PLT, platelet; ALT, alanine aminotransferase, aspartate aminotransferase; ESR, erythrocyte sedimentation rate; CRP, C-reactive protein; RF, rheumatoid factor; TNF-α, tumor necrosis factor α; IL-6, interleukin-6; ACPA, anti-citrullinated protein antibody; AKA, anti-keratin antibody; APF, anti-perinuclear factor; DAS28ESR: disease activity score 28 using erythrocyte sedimentation rate; DAS28CRP, disease activity score 28 using C-reactive protein.

Supplementary Table S4. The results of univariate analysis

**Characteristics**	**DSA28ESR**		**DAS28CRP**	
	**β (95%CI)**	***P***	**β (95%CI)**	***P***
Sex				
Male	*Ref*		*Ref*	
Female	-0.18 (-0.32, -0.03)	0.0170	-0.34 (-0.48, -0.21)	<0.0001
Age (year)	0.02 (0.01, 0.02)	<0.0001	0.02 (0.01, 0.02)	<0.0001
BMI	-0.01 (-0.03, 0.01)	0.3908	-0.00 (-0.02, 0.02)	0.9269
Smoking				
No	*Ref*		*Ref*	
Yes	0.13 (-0.13, 0.40)	0.3277	0.29 (0.03, 0.54)	0.0260
Drinking				
No	*Ref*		*Ref*	
Yes	-0.11 (-0.56, 0.35)	0.6488	0.05 (-0.39, 0.48)	0.8393
Hypertension				
No	*Ref*		*Ref*	
Yes	0.21 (0.08, 0.33)	0.0019	0.23 (0.10, 0.35)	0.0004
DM				
No	*Ref*		*Ref*	
Yes	0.19 (-0.02, 0.40)	0.0791	0.19 (-0.01, 0.39)	0.0593
CHD				

No	*Ref*		*Ref*	
Yes	0.06 (-0.18, 0.30)	0.6118	0.04 (-0.19, 0.27)	0.7429
WBC (*10^9/L)	0.11 (0.08, 0.14)	<0.0001	0.14 (0.12, 0.17)	<0.0001
Neut (*10^9/L)	0.15 (0.12, 0.18)	<0.0001	0.19 (0.16, 0.22)	<0.0001
Lymph (*10^9/L)	-0.02 (-0.12, 0.08)	0.6874	-0.01 (-0.11, 0.09)	0.8710
RBC (*10^{12}/L)	-0.46 (-0.58, -0.34)	<0.0001	-0.27 (-0.38, -0.15)	<0.0001
Hb (g/L)	-0.02 (-0.02, -0.01)	<0.0001	-0.01 (-0.01, -0.01)	<0.0001
PLT (*109/L)	0.00 (0.00, 0.00)	<0.0001	0.00 (0.00, 0.00)	<0.0001
Ln RF, IU/mL	0.14 (0.10, 0.18)	<0.0001	0.14 (0.10, 0.18)	<0.0001
Ln IL-6, pg/mL	0.17 (0.14, 0.20)	<0.0001	0.18 (0.15, 0.22)	<0.0001
Ln TNF-α, pg/mL	0.05 (-0.01, 0.11)	0.0743	0.07 (0.02, 0.13)	0.0122
Ln ALT, U/L	-0.09 (-0.19, 0.01)	0.0684	-0.07 (-0.17, 0.02)	0.1438
Ln AST, U/L	0.17 (-0.31, -0.04)	0.0104	-0.22 (-0.34, -0.09)	0.0008
Albumin (g/L)	-0.08 (-0.09, -0.07)	<0.0001	-0.08 (-0.09, -0.07)	<0.0001
Fibrinogen (g/L)	0.50 (0.45, 0.55)	<0.0001	0.49 (0.44, 0.54)	<0.0001
FAR	15.48 (14.08, 16.87)	<0.0001	15.28 (13.96, 16.60)	<0.0001
NSAIDs				
No	*Ref*		*Ref*	
Yes	-0.05 (-0.17, 0.07)	0.4251	-0.08 (-0.20, 0.03)	0.1539
Glucocorticoids				
No	*Ref*		*Ref*	

Yes	-0.17 (-0.37, 0.02)	0.0828	-0.15 (-0.33, 0.04)	0.1283
Methotrexate				
No	*Ref*		*Ref*	
Yes	-0.41 (-0.56, -0.27)	<0.0001	-0.40 (-0.55, -0.26)	<0.0001
Leflunomide				
No	*Ref*		*Ref*	
Yes	-0.23 (-0.38, -0.08)	0.0033	-0.29 (-0.44, -0.15)	<0.0001
Types of cDMARDs				
0	*Ref*		*Ref*	
1	-0.32 (-0.45, -0.19)	<0.0001	-0.37 (-0.49, -0.24)	<0.0001
2	-0.59 (-0.79, -0.40)	<0.0001	-0.56 (-0.74, -0.37)	<0.0001
3	-0.51 (-1.27, 0.26)	0.1944	-0.25 (-0.98, 0.48)	0.5016
tsDMARDs				
No	*Ref*		*Ref*	
Yes	-0.65 (-1.05, -0.25)	0.0016	-0.74 (-1.12, -0.35)	0.0002
bDMARDs				
No	*Ref*		*Ref*	
Yes	-0.22 (-0.52, 0.08)	0.1439	-0.14 (-0.42, 0.15)	0.3546

RF, TNF-α, and IL-6 were transformed using the natural logarithm prior to regression analysis. DAS28ESR, Disease activity score 28 using erythrocyte sedimentation rate, DAS28CRP, Disease activity score 28 using c-reactive protein; BMI, body mass index; DM, Diabetes Mellitus; CHD, Coronary Heart Disease; DD, disease duration; WBC, white blood cell count; Neut, neutrophil count; Lymph, lymphocyte count; RBC, red blood cell; HB, Hemoglobin; PLT, platelet; RF, rheumatoid factor; IL-6, interleukin-6; TNF-α, tumor necrosis factor

α; ALT, alanine aminotransferase; AST, aspartate aminotransferase; FAR, fibrinogen-to-albumin ratio; NSAIDs Non-Steroidal Anti-Inflammatory Drugs; cDMARDs, conventional disease-modifying antirheumatic drugs; tsDMARDs, targeted synthetic disease-modifying antirheumatic drugs; bDMARDs, biological disease-modifying antirheumatic drugs. RF, TNF-α, IL-6, ALT, and AST were transformed using the natural logarithm prior to regression analysis

Supplementary Figure S1 Correlation Analysis of FAR and its components with Traditional Inflammatory Markers ESR and CRP. ESR, Erythrocyte Sedimentation Rate; CRP, C-reactive protein; FAR, fibrinogen to albumin ratio.

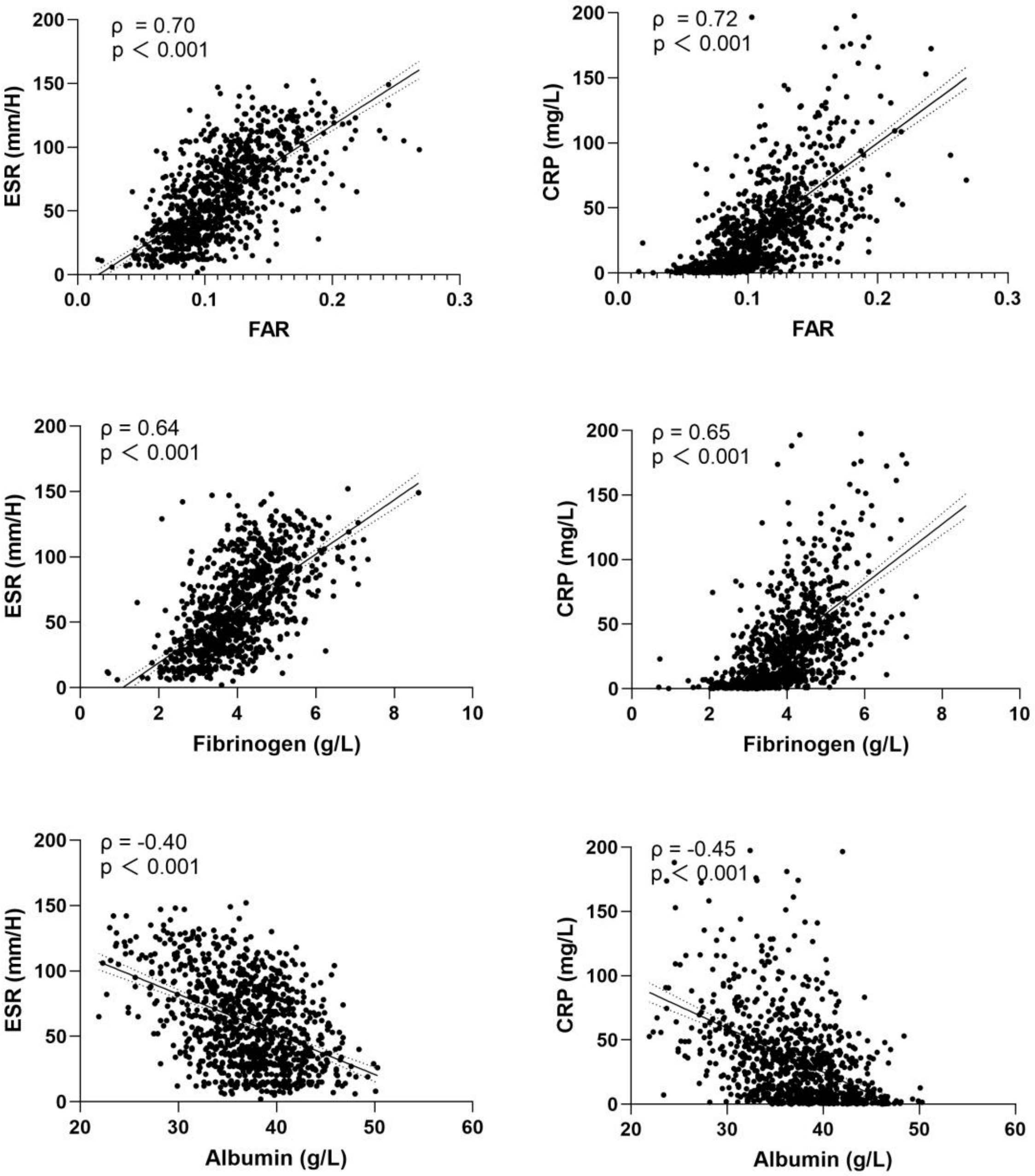

Supplementary Figure S2 The distribution of fibrinogen to albumin ratio values across the entire cohort.

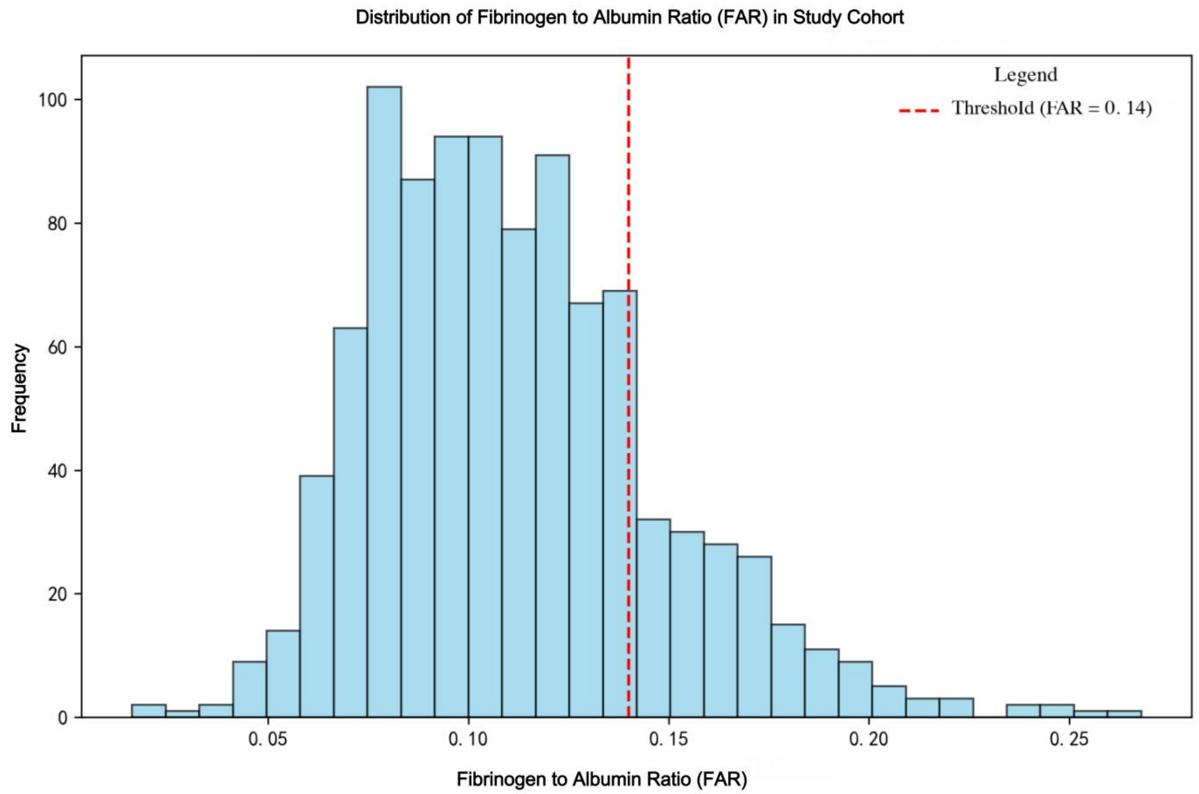

Supplementary Figure S3. The distribution of fibrinogen to albumin ratio with DAS28ESR or DAS28CRP in the entire cohort. FAR, fibrinogen to albumin ratio; DAS28ESR, Disease activity score 28 using erythrocyte sedimentation rate; DAS28CRP, Disease activity score 28 using c-reactive protein.

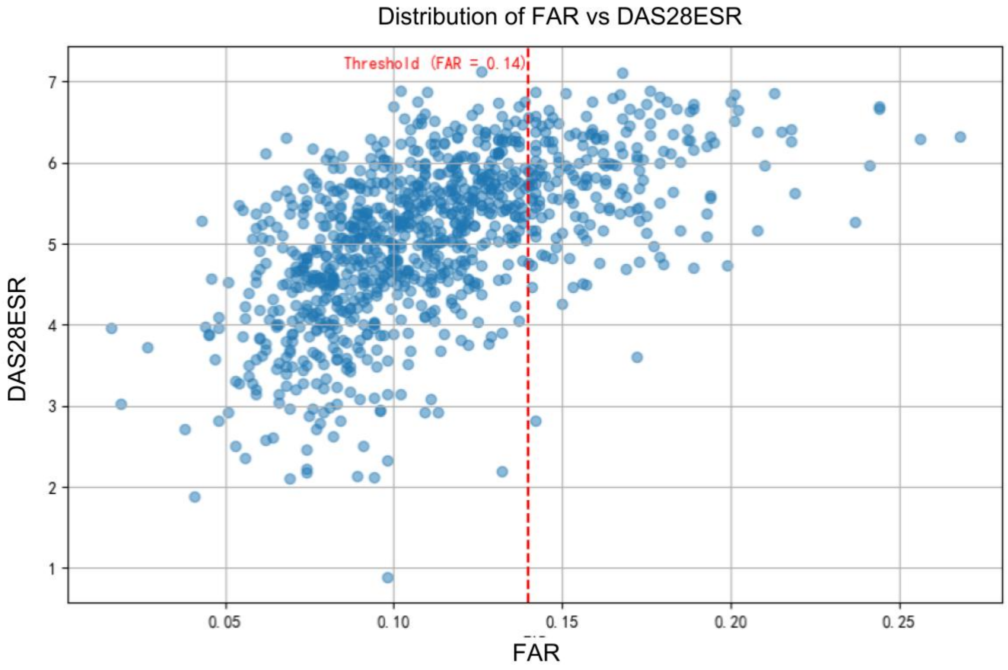

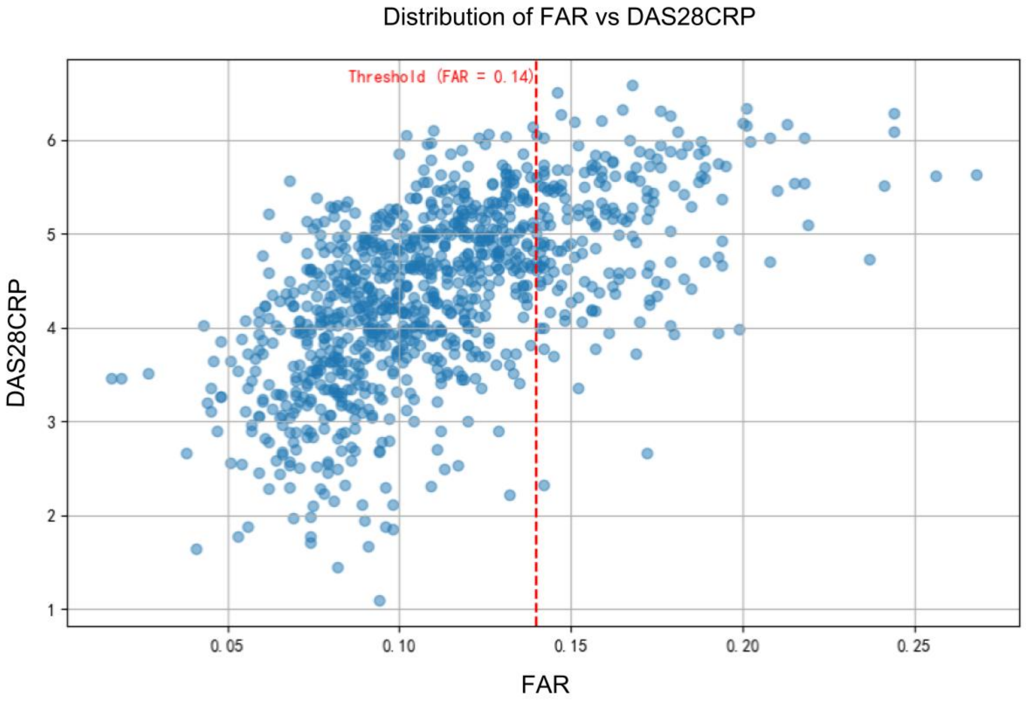

Supplement: Supplementary file 1 [file DataSheet1.pdf]
